# Dynamic Changes in Lymphocyte Populations and Their Relationship with Disease Severity and Outcome in COVID-19

**DOI:** 10.3390/ijms252211921

**Published:** 2024-11-06

**Authors:** Ákos Vince Andrejkovits, Adina Huțanu, Doina Ramona Manu, Minodora Dobreanu, Anca Meda Văsieșiu

**Affiliations:** 1 Department of Infectious Disease, George Emil Palade University of Medicine, Pharmacy, Science, and Technology, 540139 Târgu Mureș, Romania; akos.andrejkovits@umfst.ro (Á.V.A.); anca.vasiesiu@gmail.com (A.M.V.); 2 Doctoral School of Medicine, George Emil Palade University of Medicine, Pharmacy, Science, and Technology, 540139 Târgu Mureș, Romania; 3 Department of Laboratory Medicine, George Emil Palade University of Medicine, Pharmacy, Science, and Technology, 540139 Târgu Mureș, Romania; minodora.dobreanu@umfst.ro; 4 Center for Advanced Medical and Pharmaceutical Research, George Emil Palade University of Medicine, Pharmacy, Science, and Technology, 540139 Târgu Mureș, Romania; doina.manu@umfst.ro; 5 1st Infectious Disease Clinic of County Clinical Hospital, 540146 Târgu Mureș, Romania

**Keywords:** SARS-CoV-2, COVID-19, lymphocyte profiles, dynamic changes, outcome

## Abstract

Studies suggest that the dynamic changes in cellular response might correlate with disease severity and outcomes in SARS-CoV-2 patients. The study aimed to investigate the dynamic changes of lymphocyte subsets in patients with COVID-19. In this regard, 53 patients with COVID-19 were prospectively included, classified as mild, moderate, and severe. The peripheral lymphocyte profiles (LyT, LyB, and NK cells), as well as CD4+/CD8+, CD3+/CD19+, CD3+/NK and CD19+/NK ratios, and their dynamic changes during hospitalization and correlation with disease severity and outcome were assessed. We found significant differences in CD3+ lymphocytes between severity groups (*p* < 0.0001), with significantly decreased CD3+CD4+ and CD3+CD8+ in patients with severe disease (*p* < 0.0001 and *p* = 0.048, respectively). Lower CD3+/CD19+ and CD3+/NK ratios among patients with severe disease (*p* = 0.019 and *p* = 0.010, respectively) were found. The dynamic changes of lymphocyte subsets showed a significant reduction in NK cells (%) and a significant increase in CD3+CD4+ and CD3+CD8+ cells in patients with moderate and severe disease. The ROC analysis on the relationship between CD3+ cells and fatal outcome yielded an AUC of 0.723 (95% CI 0.583–0.837; *p* = 0.007), while after addition of age and SpO_2_, ferritin and NLR, the AUC significantly improved to 0.927 (95%CI 0.811–0.983), *p* < 0.001 with a sensitivity of 90.9% (95% CI 58.7–99.8%) and specificity of 85.7% (95% CI 69.7–95.2%). The absolute number of CD3+ lymphocytes might independently predict fatal outcomes in COVID-19 patients and T-lymphocyte subset evaluation in high-risk patients might be useful in estimating disease progression.

## 1. Introduction

First discovered in the 1930s, coronaviruses are one of the largest RNA viruses, which can cause a wide range of respiratory diseases, from acute respiratory tract infections to severe systemic disease [1,2]. Severe acute respiratory syndrome (SARS) was first described in November 2002 and has spread rapidly around the world, causing severe pneumonia [3].

Novel severe acute respiratory syndrome coronavirus 2 (SARS-CoV-2) infections were reported in November 2019 as an extremely pathogenic virus outbreak. Based on data reported by the World Health Organization (WHO), coronavirus disease 2019 (COVID-19) caused over 7 million deaths worldwide, of which 68,800 in Romania [4]. More than 67,000 deaths and 3 million officially reported SARS-CoV-2 infections occurred in Romania between the beginning of the pandemic and 13 November 2022 [5]. SARS-CoV-2 causes a mild or even asymptomatic disease in most patients [6]; however, in elderly patients and in those with several risk factors, the virus can lead to severe or critical COVID-19, with pneumonia, acute respiratory distress syndrome (ARDS) and multiple organ dysfunction syndrome [7]. By 2024, SARS-CoV-2 infection has transitioned to an endemic state; however, evolving variants of the virus still cause severe disease, a significant proportion of admitted patients being elderly, with multiple comorbidities [8].

Changes in the number of lymphocytes are commonly described in patients with COVID-19 [9]. SARS-CoV-2 infection can lead to immune cell alterations (both numerical and morphological), targeting T cells, B cells and natural killer (NK) cells, producing a disbalance of lymphocyte subsets and an inappropriate immune response [10].

A significant reduction of peripheral CD4+ and CD8+ T cells and NK cells has been described in patients with severe and critical COVID-19 [10,11,12]. Moreover, it has been reported that lymphocytes and their subsets, especially CD8+ T cells, might be a potential predictor of disease severity [13]. A prospective study published by Du RH et al. reported that a CD3+CD8+ T cell level of ≤75 cells/μL at admission showed high predictability for COVID-19 mortality [14]. Other studies have reported that CD8+ T cell decrease can predict severity, clinical outcome and recovery [15,16], and that a decreased CD4+ T cell count might be associated independently with admission to the ICU and the development of ARDS [17]. In patients with severe COVID-19, the virus causes extensive activation and exhaustion of NK cells to hamper host immunity, leading to important phenotypic and functional modifications. T cells and NK cells that have acquired the exhausted phenotype can be detected using exhaustion markers such as programmed cell death-1 (PD-1) or T cell immunoglobulin and mucin domain-containing-3 (TIM-3) [18,19]. In a study by Deng Z et al., NK cell numbers were increased in survivors and decreased in non-survivors [20]. In another study, the number of NK cells was increased in the first 7 days of hospitalization, corresponding with a good clinical response [21].

Moreover, several studies have shown that kinetic changes in cellular response might also be correlated with clinical outcome [21,22]. For example, a positive correlation has been described between COVID-19 improvement and the dynamic increase of white blood count (WBC), total lymphocytes, total T, CD4+ and CD8+ T cells [21,23]. Other studies reported a significant decrease of CD3+, CD4+, CD8+ cells in patients with severe or critical COVID-19 within 14 days after disease onset, as well as different normalization times for total T-lymphocyte serum levels in moderate vs. severe/critical disease (38 days vs. 49 days, respectively) [13,24]. Another study found that the disease prognosis was devastating at a total T-lymphocyte level below 500 cells/µL and a CD8+ T-lymphocyte level below 200 cells/µL [24].

These studies suggest that dynamic changes in the number of lymphocyte subsets over time might be in correlation with the prognosis of COVID-19. The detailed analysis of these dynamic changes may help predict the risk of severe disease and/or poor outcome. Therefore, the aim of this study was to investigate the dynamic modifications of lymphocyte subsets in patients with COVID-19 of different severity.

## 2. Results

### 2.1. Patient Characterization and Demographic Data

A total of 53 patients were evaluated three times during hospitalization, of which 50.9% were male, with a mean age of 69 ± 13 years, and 49.1% were female, with a mean age of 72 ± 12 years (*p* = 0.364). The majority of the patients (62.3%) had severe disease, 26.4% had moderate disease and 11.3% had mild disease. The average SpO_2_ at admission was 96% (IQR 96–98%) for mild, 95% (IQR 91–98%) for moderate and 88% (IQR 80.5–91%) for severe disease (*p* < 0.0001 for severe vs. mild/moderate disease). Most of the patients (*n* = 44, 83.1%) had at least one comorbidity, the most predominant being arterial hypertension, followed by chronic cardiac disease and obesity. Only 14 (26.4%) patients were vaccinated against SARS-CoV-2, from these patients 7 developed severe COVID-19, 3 with fatal outcome (Table 1).

The median duration of onset of symptoms to hospital admission was 7 ± 4.4 days (1–21 days). There were no significant differences between severity groups and the time of the onset of symptoms (Table 2).

### 2.2. Modifications of Lymphocyte Subsets in Relation to Disease Severity

The total number of lymphocytes, CD3+ T lymphocytes, T helper (CD4+) lymphocytes, T cytotoxic (CD8+) lymphocytes, B lymphocytes (CD19+), and NK cells, as well as the CD4+/CD8+, CD3+/CD19+, CD3+/NK and CD19+/NK ratios at admission are presented in Table 3.

We found significant differences in the total number of lymphocytes and CD3+ lymphocytes (absolute number and percentage) between groups with different disease severity. The absolute number of CD3+CD4+ and CD3+CD8+ cells was significantly decreased in the group with severe disease. There were significant differences in the percentage of NK cells between severity groups. The CD3+/CD19+ and CD3+/NK ratios were also significantly lower in patients with severe disease compared to the other two groups.

### 2.3. Dynamic Characterization of Lymphocyte Subsets in Relation to Disease Severity

Further, we evaluated the dynamic changes of the lymphocyte subsets that showed significant differences between severity groups at admission, namely NK (%), CD4+ (#) and CD8+ (#), as well as the CD3+/CD19+ and CD3+/NK ratios. The dynamics of these subsets in relation to disease severity are detailed in Table 4.

**Figure 1:**
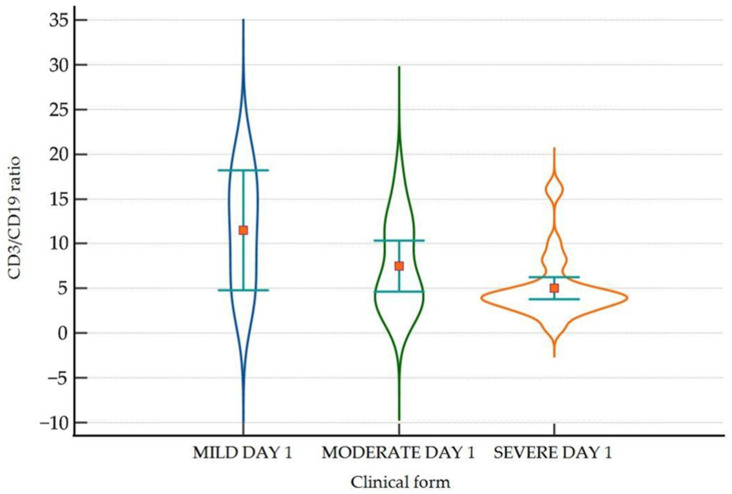
Violin-plot showing the distribution of the CD3/CD19 ratio in the three groups on day 1. CD = cluster of differentiation.

We found significant differences between groups in the average level of NK (%) on day 1 and day 5, as well as a significant decrease on day 10 compared to day 1. Regarding CD3+CD4+ and CD3+CD8+, there were significant differences between the moderate and severe groups. CD3+CD4+ was significantly lower on day 1 and day 10 in severe forms, whereas CD3+CD8+ was lower only on day 1. There was a significant reduction in the percentage of NK cells and a significant increase in the number of CD3+CD4+ and CD3+CD8+ cells during admission in patients with moderate and severe disease. We also found a significantly lower CD3+/CD19+ ratio on day 1 in severe COVID-19 (Figure 1).

**Figure 2:**
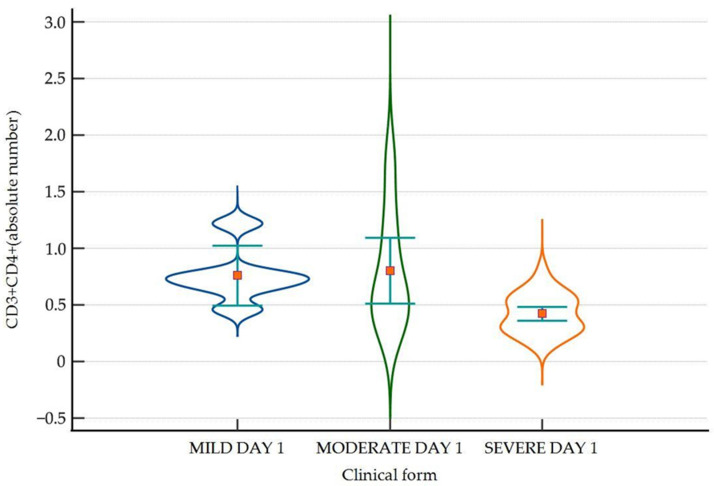
Violin-plot showing the distribution of CD3+CD4+ cells in absolute number for day 1 across disease severity, with lower levels in patients with severe disease compared to mild/moderate disease. Number of lymphocytes expressed as absolute value × 10^3^/µL. CD = cluster of differentiation.

A significantly lower level of CD3+CD8+ subsets was found in patients with severe disease compared to those with non-severe disease on day 1 (Figure 2).

Dynamic changes in lymphocyte subsets comparison in severe COVID-19 between timepoints.

**Figure 3:**
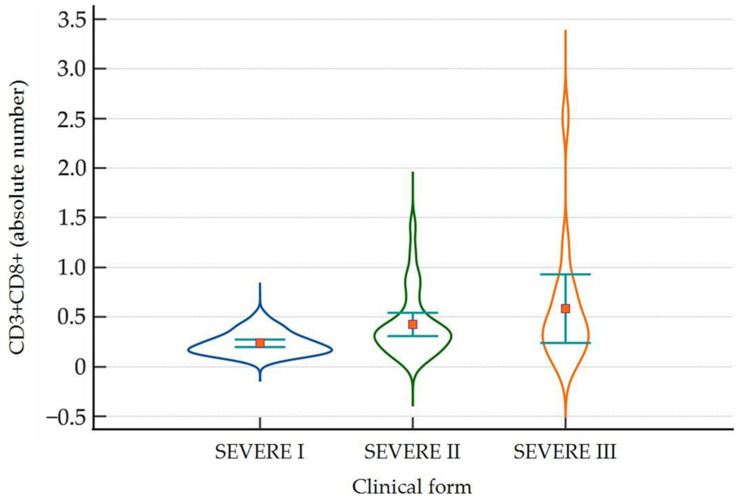
Violin-plot showing the distribution of CD3+CD8+ cells as absolute number in severe COVID-19 on day 1 (severe I), day 5 (severe II) and day 10 (severe III) of admission. Number of lymphocytes expressed as absolute value × 10^3^/µL.

A significantly lower number of CD3+CD8+ cells were found on day 1 compared to day 5 and day 10 in patients with severe COVID-19 (Figure 3).

**Figure 4:**
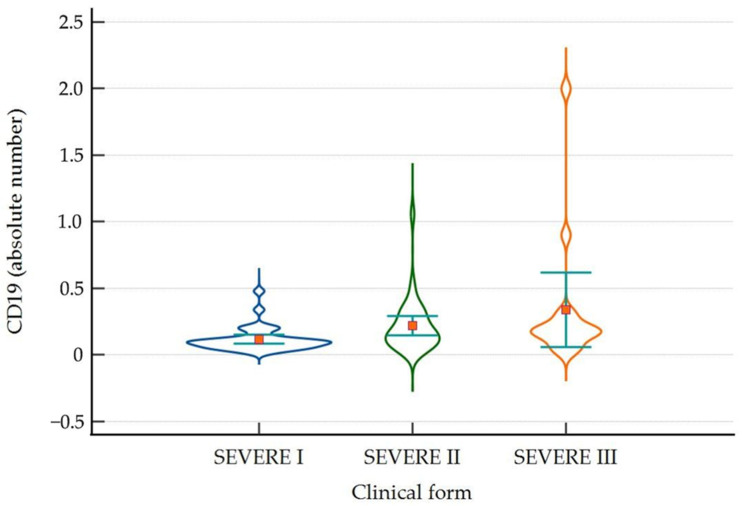
Violin-plot showing the distribution of CD19+ cells as absolute number on day 1, day 5 and day 10 in patients with severe disease. Number of lymphocytes expressed as absolute value × 10^3^/µL.

The total number of CD19+ lymphocytes was significantly lower on day 1 in severe disease, with a median of 0.100 (IQR 0.060–0.123), compared to day 5 and day 10, with a median of 0.160 (IQR 0.100–0.305) and 0.170 (IQR 0.148–0.283), respectively (*p* = 0.006) (Figure 4).

No significant differences were found between groups regarding the percentage of cells expressing surface PD-1, respectively CD3+CD4+, CD3+CD8+ and CD19+ during the hospitalization, however, in severe form the percentage of CD3+CD8+ cells expressing PD-1 was significantly lower in day 1 (Table 5).

### 2.4. Relationship Between Lymphocyte Subsets at Admission and Disease Outcomes

The distribution of lymphocyte subsets at admission by disease outcome (survivors vs. non-survivors) is presented in Table 6. We found significant differences between the two groups only in the absolute number of CD3+ and CD3+CD4+.

The percentage of cells expressing PD-1 was similar for CD3+CD4+, CD3+CD8+ and CD19+ between survivors and non-survivors (Table 7). However, for CD3+CD4+ lymphocytes, the percentage of cells expressing PD-1 was higher in non-survivors but not statistically significant (*p* = 0.351), with significantly higher median fluorescence intensity (MIF) of the PD-1 marker on the surface of CD4+ cells for non-survivors (*p* = 0.007). The same pattern was observed for the median fluorescence expression of the PD-1 marker on CD3+CD8+ lymphocytes, with higher expression in non-survivors (median 618, IQR 577–674) compared to survivors (median 574, IQR 547–609) (*p* = 0.048) (Table 7).

A univariate logistic regression analysis was used to estimate the influence of selected independent variables (age, gender, CD3+ (#), CD3+CD4+ (#), CD3+CD8+ (#), NK (%) and CD19+ (#)) on fatal outcome (Table 8). The analysis has shown that only the total number of CD3+ (#) has a predictive capacity.

In the multiple logistic regression, we considered age, gender, and total CD3+ (#). None of these variables were found to predict fatal outcome (Table 9).

In the ROC analysis, we found a CD3+ cut-off value of 0.520 × 10^3^/µL for fatal evolution, with an area under the curve (AUC) of 0.723 (95% CI 0.583–0.837) (*p* = 0.007).

**Figure 5:**
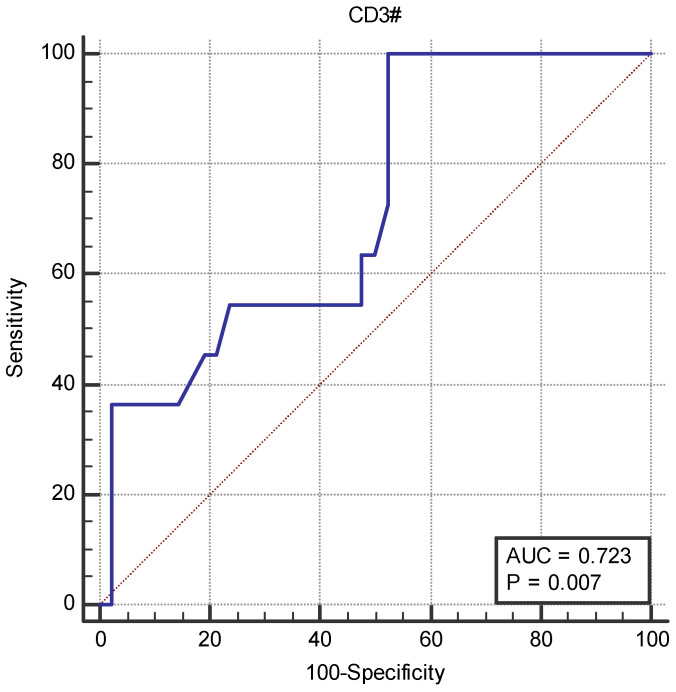
ROC analysis and AUC for CD3+ cells and fatal outcome.

For this cut-off value, the associated Youden index was 0.476, with a sensitivity of 100% (95% CI 71.5–100%) and a specificity of 47.62% (95% CI 32–63.6%) (Figure 5).

**Figure 6:**
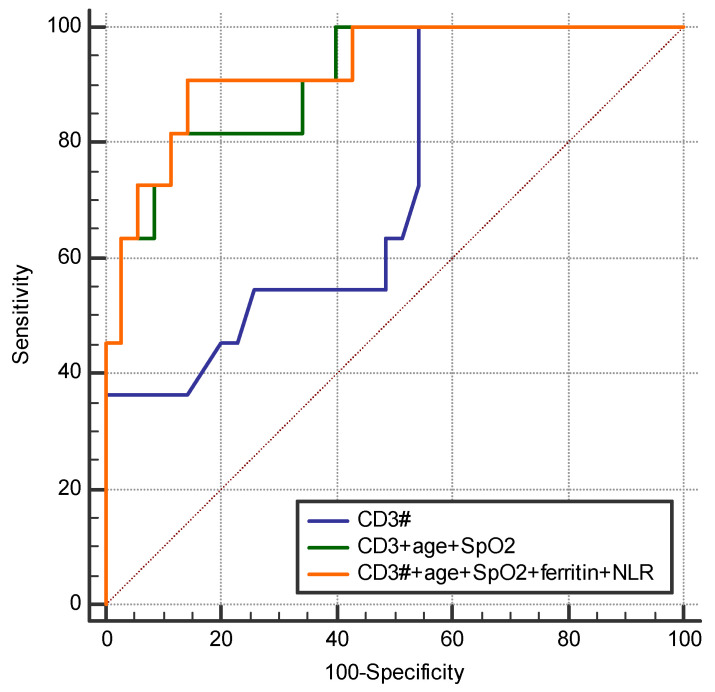
Comparison of ROC curves for three models (model 1 = CD3+) and models with multiple biomarkers (clinical and laboratory) for fatal outcome prediction.

To increase the sensibility and specificity, multiple models were created using biomarkers that are commonly measured in COVID-19 patients. By incorporating the patient’s age and the SpO_2_ measured at admission, AUC was 0.909 (95% CI 0.795–0.971) with significantly improved sensitivity 81.8% (95% CI: 48.2–97.7) and specificity 90.0% (95%CI: 76.3–97.2), *p* < 0.0001. After the addition of ferritin and neutrophil to lymphocyte ratio (NLR), a cost-effective biomarker derived from the complete blood count (CBC) the AUC was 0.927 (95%CI 0.811–0.983), with a sensitivity of 90.9% (95% CI 58.7–99.8%) and specificity of 85.7% (95% CI 69.7–95.2%) *p* < 0.001 (Figure 6).

## 3. Discussion

Lymphocytes and their subsets have an essential role in maintaining normal immune functions. Lymphopenia was recognized as a characteristic modification in patients with COVID-19, especially in those with a severe form of the disease [25]. Shouman et al. describe four possible mechanisms: metabolic changes in the context of uncontrolled cytokine production, dysregulated hematopoiesis by a direct or indirect viral mechanism, lipid raft by T cell activation can offer viral entrance, or direct viral infection through receptors. It is also possible that these mechanisms work together to facilitate viral infection [26]. Similarly to other studies [10,22,27] we found significantly lower lymphocyte numbers in patients with moderate and severe COVID-19. These modifications underline the protective function of T lymphocytes against SARS-CoV-2, but could also be correlated with poor prognosis. Further, we discuss the modifications of lymphocyte subsets.

A reduced number of T-helper (CD3+CD4+), cytotoxic (CD3+CD8+) and NK cells were observed on day 1 of admission in patients with severe disease compared to those with mild or moderate disease. Our findings also confirm the presence of lymphopenia, particularly in the CD4 + and CD8+ subsets, described by other studies [22,23,27].

By examining the dynamic changes of lymphocyte subsets, we observed significant differences between severity groups. NK (%) was significantly decreased on day 1 and day 5 in severe forms, with a significant difference between patients with severe vs. mild/moderate disease. Given that a normal NK cell response can improve SARS-CoV-2 infection control by direct anti-SARS-CoV-2 activity, this significant decrease might suggest inflammation and intensive cell apoptosis [28]. Some authors believe that the decrease in peripheral NK levels might be attributed to a relocation of NK cells from the blood to the airway epithelium [29,30], whereas others suggest that it may be in correlation with the magnitude of the systemic inflammation or a consequence of direct, viral-induced apoptosis [28]. Leem et al. found that in addition to a reduction in their number, the function of NK cells is also impaired [31], demonstrating a significantly reduced cytotoxic function in COVID-19. Further research is needed to assess the reduced number, subtypes, and functional impairments of these lymphocyte subtypes.

Comparing NK cell levels between different severity groups over the course of admission, there were no significant differences in mild form; however, we noted a significant decrease of NK cells in patients with moderate COVID-19. In severe forms, NK (%) was significantly lower on day 5 than on day 1, followed by a slight recovery on day 10. As highlighted by Di Vito et al., this decrease is probably directly correlated with disease severity in the acute phase [32]. Moreover, it has been described that NK and T cell counts are restored after the acute phase of COVID-19, whereas in patients with a poor outcome a gradual decrease in the number of NK cells occurs [33,34]. We did not observe this phenomenon in the dynamics of the absolute number of NK cells; however, the percentage of NK cells showed an important decrease in severe cases and a slight recovery on day 10, but with no significant differences between survivors and non-survivors. This delayed recovery in severe COVID-19 compared to non-severe forms has also been described by other studies [31].

Usually, CD4+ and CD8+ T lymphocytes contribute to virus control by secreting perforins, granzymes and interferons, eliminating the infected cells [35,36]. In our study, the dynamics of T helper (CD3+CD4+) and T cytotoxic (CD3 +CD8+) cells showed significant differences between severity groups only on day 1 of admission, with a lower average in severe cases. This could indicate an impaired cellular immunity in the early stage of severe COVID-19. According to Sukrisman L. et al. [10] these modifications are linked to substantial inflammation and tissue injury. In moderate and severe forms, we observed a significantly higher number of CD3+CD4+ and CD3+CD8+ cells on day 10 compared with day 1, which suggests a positive correlation between decreased T helper and T cytotoxic cell numbers and disease severity.

In a longitudinal analysis of SARS-CoV-2-specific CD4+ and CD8+ T cells in patients admitted to the ICU it was observed that there are potential variations in T-cell responses depending on disease severity [37]. It has also been demonstrated that certain T cell phenotypes are associated with different kinetics of the immune response in patients with COVID-19 [37,38]. However, the patients in our study were in different phases of the disease, which could explain the differences in the results.

Studies have shown that elevated levels of the PD-1 marker reflect T lymphocyte exhaustion and are associated with increased severity of SARS-CoV-2 infection [39]. In this study, the expression of this marker on CD3+CD4+, CD3+CD8+ and CD19+ cells were similar between survivors and non-survivors. Studies have also shown a relationship between PD-1 expression and disease severity [19,40]. We did not find any correlation of this kind, which may be explained by the relatively small number of patients included in our study. However, the median fluorescence expression of PD-1 on CD3+CD8+ lymphocytes was significantly higher in non-survivors.

Guan et al. [41] found that leukocytes, neutrophils and CD3+CD4−CD8− cells had great diagnostic efficacy for SARS-CoV-2 infection, distinguishing severe cases from mild ones. In our study, CD3+ cells were correlated with disease severity and served as a predictor of mortality. The ROC analysis of CD3+ lymphocytes among non-survivors yielded an AUC of 0.723, which is similar to the values reported by other studies [42,43].

Very recently, Shouman et al. reviewed possible mechanisms behind lymphocyte depletion in COVID-19 patients and underlined that the occurrence of lymphopenia is a sign of an unfavorable prognosis. Many mechanisms seem to be involved in severe lymphocyte depletion in SARS-CoV-2 infection, mostly related to cytokine storm or increased apoptosis [26]. Moreover, lactic acidosis negatively impacts cell metabolism suppressing lymphocyte proliferation or increasing apoptosis [26]. Also, the overproduction of interleukin 6 (IL-6) is considered one of the major suppressors of lymphopoiesis [26], and plasma level of IL-6, associated with severe disease, is a predictor of ICU transfer and fatal outcome [44]. Increased expression of PD-1 on the cell’s surface was considered a marker of T-cell exhaustion. Although in our study we did not find differences in the percentage of cells expressing PD-1 across the disease severity nor the time-points, the intensity of the PD-1 expressed on the surface of CD3+CD4+ and CD3+CD8+ was significantly higher in patients with fatal outcomes.

The analysis of lymphocytes could provide more insights into the inflammatory status and clinical disease progression, and mapping the lymphocyte subsets allows clinicians to differentiate between COVID-19 and COVID-like patients. In this line, the study performed by Balzanelli et al. who deeply analyzed the lymphocyte subsets in COVID-19 and non-COVID patients revealed a profound alteration in lymphocyte population in case of SARS-CoV-2 infection, with low B cells, low Treg, high T cells and high T-NK cells [11]. Also, the evaluation of the immune response in patients with long COVID, as well as the shift to later activation of active T cells (CD3+DR+) or CD8+ could represent the early appearance of the adaptive immune response [11]. The study performed by Dai and collab. analyzed the relationship between lymphocyte subtypes and proinflammatory cytokine IL-6 in patients with SARS-CoV-2 infection, and found that for those with normal IL-6 levels, the T cells, including naïve and central memory T cells, T reg, NK cells, effectory CD8+ cells, and class-switched memory B cells were moderately increased [12]. For patients with less than a 30-fold increase in IL-6, the class unswitched memory B cells, marginal B cells, NKT cells, naïve Treg, and differentiated T CD4 cells were increased, while for patients with more than a 30-fold increase in IL-6 plasma levels, except for the plasmablast and Treg, almost all lymphocytes subsets were decreased [12]. Additionally, by performing the single-cell RNA-sequencing (scRNA-seq), the authors were able to characterize the cell-specific genes for various lymphocyte subsets, and to compare results with those in healthy control [12]. Our findings suggest that T-lymphocyte subset evaluation, especially CD3+, could be a promising predictor of disease progression.

## 4. Materials and Methods

This observational prospective study included 56 adult patients with COVID-19 admitted to the 1st Infectious Disease Clinic County Hospital of Targu Mures, Romania between December 2021 and February 2022, during the 4th and 5th waves of the pandemic in Romania. Peripheral lymphocyte profiles and their dynamic changes were evaluated during hospitalization. The diagnosis of SARS-CoV-2 infection was confirmed by positive reverse transcription polymerase chain reaction (RT-PCR) test. Disease severity was established based on the clinical signs and symptoms, oxygen saturation (SpO_2_) on room air, respiratory rate, and the presence of respiratory distress syndrome or organ dysfunction, and was classified as mild, moderate and severe, as reported previously [45].

Inclusion criteria were the following: positive RT–PCR test for SARS-CoV-2, admission to the Infection Disease Clinic Nr. I. of Târgu Mureș, willingness to participate in the study, age > 18 years, without immunodeficiencies (e.g., HIV/AIDS, immunosuppressive therapy, malignancies) or pregnancy. Of the 131 patients admitted in the study period, 56 have agreed to participate in the study. Three patients were excluded, one with wrong classification and two as technical outliers.

We assessed lymphocyte levels (absolute number and percentage) and dynamic changes in peripheral lymphocyte profiles (CD3+, CD4+, CD8+, CD19+ T cells, B cells and NK cells), and calculated CD4+/CD8+, CD3+/CD19+, CD3+/NK and CD19+/NK ratios during the acute phase of SARS-CoV-2 infection, when cellular immunity is the most active. Therefore, the dynamic changes of lymphocyte profiles were assessed during the first 10 days of admission. The results were correlated with disease severity and outcome (survivors and non-survivors).

### 4.1. Blood Test Analysis

We selected three timepoints for the analysis, for a better characterization of the lymphocyte changes: day 1, day 5 and day 10 of admission. For patients with shorter hospitalization periods due to hospital discharge or death, the blood analysis was performed accordingly. Blood collection was performed in the morning into two EDTA tubes, one for complete blood count and the other for lymphocyte phenotyping. The complete blood count analysis was performed on a Sysmex XS-800i analyzer (Sysmex, Kobe, Japan), and the differentiated leucocyte formula was recorded both in percentages (%) and absolute numbers (#).

### 4.2. Immunophenotyping and Characterization of the Lymphocyte Subsets

The immune characterization of the lymphocyte population and the expression of PD-1 on their surface was performed using a BD FACSAria III flow cytometer. The cytometer configuration and set-up, the fluorochromes, and the specific antibodies used for labeling protocol are described below (Table 10).

For the analysis of lymphocytes by flow cytometry, 50 μL of whole blood were incubated with the mixture of antibodies, previously prepared in staining buffer (BD cat. no. 554656). The panel of antibodies was built based on the brightness of the fluorochromes, the density and the co-expression of antigens on a certain leukocyte subtype, and to avoid fluorochrome emission spillover (Table 10). The optimal antibody concentrations were assessed based on the number of cells fluorescently labeled with specific antibodies. After incubation with antibodies, the samples were treated for red blood cell lysis with BD Pharm Lyse ™ lysing solution (BD cat. no. 555899), followed by a washing step with PBS. The data generated by the labeled cells were acquired and analyzed using a BD FACSAria III cytometer (Becton, Dickinson and Company BD Biosciences, San Jose, CA, USA) and BD FACSDiva^TM^ version 8.0.1 software. BD setup and tracking beads (BD cat. no. 655050) were used to prevent variations in the acquisition parameters between runs.

**Figure 7:**
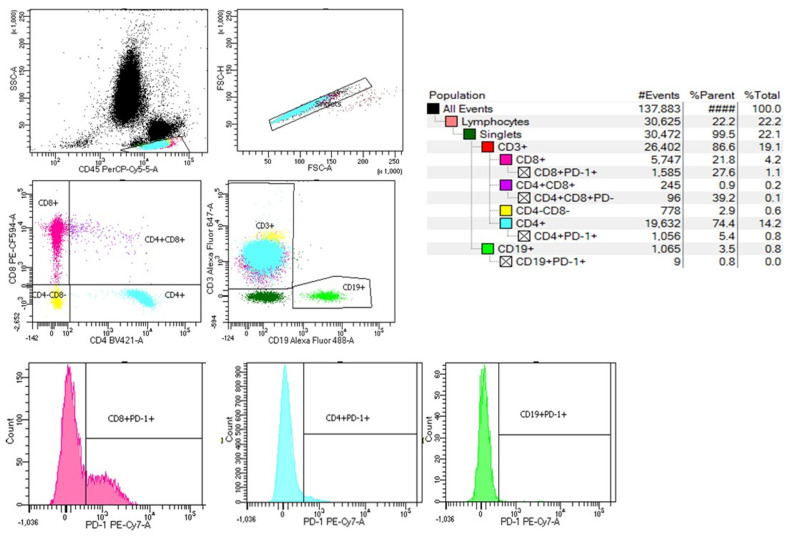
Example of a histogram and gating strategy for lymphocyte subpopulations.

During the flow cytometry acquisition, at least 30,000 singlets were acquired for each blood sample, and white blood cells were gated in CD45/SSC. From lymphocyte singlets, CD3+ and CD19+ lymphocyte subpopulations were gated and dot plots for CD3+CD8+ and CD3+CD4+ cells were drawn. The expression of PD-1 on each lymphocyte subset was analyzed as median fluorescence intensity (MFI) on monoparametric histograms (Figure 7).

The gating strategy and evaluation of NK cells has been detailed elsewhere [46]. Briefly, white blood cells were gated in CD45/SSC and differentiated expression of CD16 and CD56 markers.

### 4.3. Statistical Analysis

Statistical analysis was performed using MedCalc^®^ Statistical Software version 20.104 (MedCalc^®^ Software Ltd., Ostend, Belgium; https://www.medcalc.org). Comparability between groups was performed depending on data distribution. Continuous variables with normal distribution were assessed using Student’s t-test and expressed as mean ± standard deviation (SD). Data with non-gaussian distribution were assessed using the Mann–Whitney test and expressed as median and interquartile range (IQR). The normality of data was tested with the Kolmogorov–Smirnov and Shapiro–Wilk tests. Categorical variables were compared the chi-squared test or Fisher’s exact test, and expressed as numbers and percentages. For dynamic changes of cell populations, the Kruskal–Wallis test with Dunn’s post-hoc analysis was performed, and a graphical representation using violin-shaped plots was used for between-moments comparisons of the most significant parameters. Receiver operative characteristic (ROC) curves analysis for CD3+ lymphocytes and multiple biomarkers were performed and assessed the relationship with fatal outcome. A univariate logistic regression analysis was used for the estimation of the influence of selected independent variables on disease-related outcomes. Multiple logistic regression analysis was performed regarding fatal outcome prediction. A *p*-value of ≤0.05 was considered statistically significant.

## 5. Conclusions

We found a significant decrease in the number of CD3+CD4+ and CD3+CD8+ lymphocytes and the percentage of NK cells in patients with severe COVID-19 on day 1 of admission. These patients also had lower CD3+/CD19+ and CD3+/NK ratios. We found a positive correlation between disease severity and the number of CD3+, CD3+CD4+ and CD3+CD8+ lymphocytes, as well as a correlation between the significant decrease of CD3+ and CD3+CD4+ cells and fatal outcome.

CD3+CD4+ cell levels were the lowest on day 5, which was associated with poor recovery in patients with severe disease. CD3+CD8+ cell levels were also significantly lower among patients with severe disease, with an increasing trend in each severity group. The CD3+/CD19+ ratio was significantly lower on day 1 among patients with severe disease and also followed an increasing trend during admission. Significantly higher median fluorescence intensity (MIF) of the PD-1 marker on the surface of CD4+ and CD8+ cells for non-survivors. Our findings suggest that the absolute number of CD3+ lymphocytes can be used as an independent predictor for the fatal outcome, while the combination of CD3+ with usual biomarkers (ferritin and NLR), age and SpO_2_ provides an improved sensibility and specificity. Implementing a T-lymphocyte subset evaluation in high-risk patients might be useful for patient stratification and to predict disease progression.

## 6. Strengths and Limitations

The main strength of the study is that it assessed the dynamic changes occurring in lymphocyte subsets in the first 10 days of infection, in the acute phase of the disease. However, it also has a few limitations, the most important being the relatively low number of included patients. In addition, more than 60% of included patients had severe COVID-19, which may have contributed to an unbalanced study population. This can be explained by the relatively low number of patients admitted to our unit in the study period and the even lower number of patients who were willing to participate in the study. Another limitation is represented by the relatively low sensitivity and specificity values yielded by the ROC analysis, which warrant further study of the relationship between lymphocyte subset dynamics and disease prognosis. Furthermore, this study did not take into consideration the vaccination status of the patients and its potential effect on cellular immunity.

## Figures and Tables

**Table 1:** Demographic and clinical data of the patients.

Variables	Value
**Total number of patients**	53
**Male**	27 (50.9%)
**Female**	26 (49.1%)
**Severity**	
**Mild**	6 (11.3%)
**Moderate**	14 (26.4%)
**Severe**	33 (62.3%)
**Comorbidities**	
**No comorbidities**	9 (16.9%)
**Hypertension**	39 (73.5%)
**Chronic cardiac disease**	31 (58.5%)
**Chronic kidney failure**	7 (13.2%)
**Chronic hepatopathies**	3 (5.6%)
**Pulmonary fibrosis**	2 (3.7%)
**Chronic obstructive pulmonary disease/pulmonary fibrosis**	9 (16.9%)
**Obesity**	16 (30.2%)
**Dyslipidemia**	9 (16.9%)
**Vaccinated**	14 (26.4%)
**Disease outcome**	
**Fatal outcome**	11 (20.7%)
**Survivors**	42 (79.2%)

All data expressed as *n* (%).

**Table 2:** The median duration of symptom onset to hospital admission.

Variables	Mild (*n* = 6)	Moderate (*n* = 14)	Severe (*n* = 33)	*p* Value
**Onset of symptoms to hospital admission**	4 (2–6)	7.5 (2–11)	7 (3.7–10)	0.547
	**Survivors (*n* = 42)**		**Non-survivors (*n* = 11)**	
**Onset of symptoms to hospital admission**	7 (3–9)		9 (3.75–12.25)	0.372

Median duration of onset to admission, expressed as days (IQR). All *p* values from the Kruskal-Wallis test.

**Table 3:** Average lymphocyte subpopulations and ratios by disease severity at admission.

Variables	Mild (*n* = 6)	Moderate (*n* = 14)	Severe (*n* = 33)	*p* Value
**Lymphocytes (#)**	1.11 (0.94–1.64)	1.09 (0.68–1.58)	0.74 (0.57–0.83)	** 0.001 **
**Lymphocytes (%)**	17.9 (14.7–26.5)	14.1 (10.5–19.1)	7.7 (5.8–11.7)	** <0.0001 **
**CD3+ (#)**	0.85 (0.73–1.25)	0.55 (0.39–1.06)	0.40 (0.30–0.51)	** 0.001 **
**CD3+ (%)**	74.45 (68.0–82.3)	64.35 (48.30–66.90)	56.30 (48.22–66.45)	** 0.013 **
**CD3+CD4+ (#)**	0.73 (0.65–0.77)	0.59 (0.44–1.20)	0.42 (0.29–0.55)	** <0.0001 **
**CD3+CD4+ (%)**	64.55 (62.8–66.3)	69.8 (59.6–77.4)	63.5 (44.6–72.42)	0.221
**CD3+CD8+ (#)**	0.32 (0.27–0.60)	0.31 (0.17–0.46)	0.23 (0.15–0.31)	** 0.048 **
**CD3+CD8+ (%)**	30.6 (28.8–31.9)	25.1 (18.3–31.1)	30.8 (22.6–40.45)	0.288
**CD4+/CD8+**	2.11 (1.99–2.39)	2.61 (1.71–4.25)	2.12 (1.25–3.06)	0.333
**NK (#)**	0.105 (0.07–0.16)	0.205 (0.14–0.29)	0.14 (0.07–0.18)	0.100
**NK (%)**	9.75 (8.2–11.2)	23.25 (11.7–27.3)	18.1 (11.87–27.3)	** 0.040 **
**CD19+ (#)**	0.09 (0.08–0.11)	0.09 (0.03–0.21)	0.10 (0.06–0.12)	0.924
**CD19+ (%)**	6.8 (4.3–15.1)	10.2 (7.1–14.5)	13.2 (10.42–16.27)	0.077
**CD3+/CD19+**	11.93 (4.5–16.95)	5.27 (3.32–11.58)	4.09 (3.05–5.19)	** 0.019 **
**CD3+/NK**	7.47 (7.16–7.68)	2.38 (1.98–5.72)	2.60 (1.99–5.43)	** 0.010 **
**CD19+/NK**	0.92 (0.38–1.59)	0.47 (0.36–0.98)	0.70 (0.42–1.40)	0.366

Absolute numbers are expressed as cells × 10^3^/µL. All data expressed as median (range). All *p* values from the Mann–Whitney test. CD = cluster of differentiation, NK = Natural Killer

**Table 4:** Dynamic changes in lymphocyte subsets and ratios by disease severity.

Timepoint	Mild (*n* = 6)	Moderate (*n* = 14)	Severe (*n* = 33)	*p* Value
**NK (%)**				
**Day 1**	9.75 (8.20–11.20)	23.25 (11.70–27.30)	18.10 (11.85–27.30)	**0.041**
**Day 5**	9.90 (6.80–13.70)	17.40 (14.55–20.05)	9.75 (6.50–15.40)	**0.024**
**Day 10**	–	7.35 (4.55–10.80)	10.10 (7.07–13.07)	0.193
***p* value**	0.936	**0.022**	**<0.001**	
**CD3+CD4+ (#)**				
**Day 1**	0.73 (0.65–0.77)	0.59 (0.44–1.20)	0.42 (0.29–0.55)	**<0.0001**
**Day 5**	0.78 (0.68–1.15)	0.56 (0.41–0.86)	0.55 (0.28–0.83)	0.072
**Day 10**	–	1.83 (1.33–1.97)	0.83 (0.34–1.05)	**0.045**
***p* value**	0.574	**0.024**	**0.035**	
**CD3+CD8+ (#)**				
**Day 1**	0.32 (0.27–0.60)	0.31 (0.17–0.46)	0.23 (0.15–0.30)	**0.048**
**Day 5**	0.47 (0.34–0.73)	0.35 (0.20–0.71)	0.34 (0.19–0.53)	0.410
**Day 10**	–	0.66 (0.56–1.20)	0.43 (0.25–0.71)	0.121
***p* value**	0.377	**0.040**	**0.005**	
**CD3+/CD19+**				
**Day 1**	11.93 (4.50–16.95)	5.27 (3.32–11.58)	4.09 (3.05–5.19)	**0.019**
**Day 5**	6.93 (5.84–17.07)	4.84 (3.32–9.58)	4.27 (2.51–5.66)	0.060
**Day 10**	–	4.02 (2.63–14.18)	4.40 (2.42–6.05)	0.961
***p* value**	1.00	0.817	0.933	
**CD4+/CD8+**				
**Day 1**	2.11 (1.99–2.39)	2.74 (1.92–4.19)	2.12 (1.27–3.06)	0.247
**Day 5**	2.06 (1.43–2.57)	1.69 (0.97–3.12)	1.92 (0.83–3.09)	0.992
**Day 10**	–	2.99 (1.54–3.45)	1.49 (0.87–3.45)	0.880
***p* value**	0.748	0.376	0.709	

**Table 5:** Dynamic changes in the percentage of cells expressing surface PD-1 for CD3+CD4+, CD3+CD8+ and CD19+ across disease severity.

Timepoint	Mild (*n* = 6)	Moderate (*n* = 14)	Severe (*n* = 33)	*p* Value
**CD3+CD4+ PD-1+ (%)**				
**Day 1**	6.35 (4.4–19.4)	9.5 (3.1–13.1)	9.5 (1.8–28.9)	0.544
**Day 5**	6.25 (3.5–10.8)	8.05 (2.7–16.00)	8.20 (2.4–27.3)	0.458
**Day 10**	-	6.8 (3.9–11.0)	8.9 (3.1–55.7)	0.250
***p* value**	0.772	0.615	0.452	
**CD3+CD8+ PD-1 (%)**				
**Day 1**	10.95 (5.4–52.5)	14.85 (2.9–43.1)	15.1 (2.2–48.8)	0.633
**Day 5**	10.75 (6.7–23.8)	19.35 (6.2–30.4)	18.9 (3.6–42.7)	0.156
**Day 10**	–	13.6 (9.5–28.1)	25.00 (5.9–67.6)	0.119
***p* value**	0.390	0.478	**0.029**	
**CD19+PD-1 (%)**				
**Day 1**	0.6 (0.2–1.3)	0.85 (0.3–2.8)	0.6 (0.0–3.5)	0.411
**Day 5**	0.7 (0.3–1.6)	0.65 (0.3–2.1)	0.6 (0.0–2.3)	0.725
**Day 10**	–	0.7 (0.5–0.9)	0.5 (0.0–3.9)	0.499
***p* value**	0.686	0.584	0.655	

**Table 6:** Lymphocyte subsets (absolute numbers and percentages) and ratios on admission, compared by outcome.

Parameter	Survivors (*n* = 42)	Non-Survivors (*n* = 11)	*p* Value
**CD19+ (%)**	13.05 (7.50–14.6)	13.1 (10.47–21.15)	0.130
**CD19+ (#)**	0.09 (0.05–0.19)	0.10 (0.07–0.150)	0.851
**CD3+ (%)**	62.4 (52.2–68.0)	53.7 (38.7–64.1)	0.091
**CD3+ (#)**	0.46 (0.34–0.77)	0.32 (0.22–0.48)	**0.023**
**CD3+CD4+ (%)**	65.2 (52.9–73.3)	66.9 (39.3–72.2)	0.510
**CD3+CD4+ (#)**	0.53 (0.33–0.70)	0.42 (0.22–0.52)	**0.038**
**CD3+CD8+ (%)**	29.6 (22.6–34.9)	25.2 (23.3–51.6)	0.476
**CD3+CD8+ (#)**	0.25 (0.16–0.36)	0.23 (0.15–0.31)	0.531
**NK (%)**	18.15 (10.7–26.4)	15.4 (11.8–29.2)	0.685
**NK (#)**	0.15 (0.10–0.22)	0.13 (0.07–0.19)	0.339
**CD3+/CD19+**	4.50 (3.81–10.3)	3.64 (2.44–5.23)	0.056
**CD4+/CD8+**	2.24 (1.70–3.24)	2.68 (0.77–3.05)	0.539
**CD19+/NK**	0.64 (0.38–1.27)	0.85 (0.41–1.41)	0.496
**CD3+/NK**	3.20 (2.04–6.48)	2.60 (1.59–3.98)	0.386

Absolute numbers (#) are expressed as cells × 10^3^/µL. All data expressed as median (range). All *p* values from the Mann–Whitney test. CD = cluster of differentiation, NK = Natural Killer.

**Table 7:** The percentage of cells expressing surface PD-1 and the median fluorescence intensity (MIF) on admission were compared by outcome.

Parameter	Survivors (*n* = 42)	Non-Survivors (*n* = 11)	*p* Value
**CD3+CD4+ PD-1+ (%)**	9.25 (6.9–12.2)	12.2 (6.55–17.97)	0.351
**CD3+CD8+ PD-1 (%)**	13.85 (10.2–20.3)	15.6 (14.02–26.55)	0.207
**CD19+PD-1 (%)**	0.7 (0.5–1.1)	0.5 (0.3–0.97)	0.294
**CD3+CD4+PD-1+ (MIF)**	645.0 (600.0–679.0)	765.0 (658.0–843.75)	**0.007**
**CD3+CD8+PD-1+ (MIF)**	574.0 (547.0–609.5)	618.0 (577.5–674.5)	**0.048**
**CD19+PD-1+ (MIF)**	517.5 (476.0–606.0)	587.0 (501.75–645.25)	0.297

All data expressed as median (range). All *p* values from the Mann–Whitney test. CD = cluster of differentiation, MIF = median fluorescence intensity; PD-1 = Programmed cell death protein 1.

**Table 8:** Univariate logistic regression analysis for fatal outcome.

Variable	Coefficient	Std. Error	Wald	*p* Value
**Age**	0.039678	0.029584	1.7988	0.179
**Gender = “F”**	0.27763	0.67979	0.1668	0.683
**CD3+ (#)**	–4.66242	2.27864	4.1867	**0.040**
**CD3+CD4+ (#)**	–3.69402	1.97802	3.4877	0.061
**CD3+CD8+ (#)**	–2.05821	2.52196	0.6660	0.414
**CD19+ (#)**	4.54248	3.58531	1.6052	0.205
**NK (%)**	0.025176	0.030746	0.6705	0.412

**Table 9:** Multiple logistic regression analysis for fatal outcome.

Variable	Coefficient	Std. Error	Wald	*p* Value
**Age**	0.025594	0.032664	0.6140	0.4333
**Gender = “F”**	0.18059	0.75612	0.05704	0.8112
**CD3+ (#)**	–4.50801	2.32120	3.7718	0.0521
**Constant**	–1.31977	2.59104	0.2594	0.6105

**Table 10:** Parameter specifications and BD FACSAria III flow cytometer configuration used for data acquisition during the study protocol.

Excitation LASER	Fluorochrome	Band-Pass Filters (nm)	Specificity	Mouse Antibody
**Violet (405 nm)**	BD Horizon™ BV421	450/40	Human CD4	BD 562424
**Blue (488 nm)**	BD Pharmingen™ Alexa Fluor 488	530/30	Human CD19	BD 557697
	BD Horizon™ PE-CF594	616/20	Human CD8α	BD 562282
	BD Pharmingen™ PerCp Cy 5.5	695/40	Human CD45	BD 564105
	BD Pharmingen™ PE-Cy7	780/60	Human PD-1	BD 561272
	BD Pharmingen™ PE	575/26	Human CD56	BD 555516
**Red (633 nm)**	BD Pharmingen™ Alexa Fluor 647	660/20	Human CD3ε	BD 566686
	BD Pharmingen™ Alexa Fluor^®^ 700	730/45	Human CD16	BD 560713

CD = cluster of differentiation; BV = Brilliant Violet; PE-CF594 = Phycoerythrin-Cyanine-based Fluorochrome 594; PE-Cy7 = Phycoerythrin-Cyanine 7; PerCP-Cy 5.5 = Peridinin Chlorophyll Protein Complex-Cyanine 5.5.

## Data Availability

Data supporting the results of this article will be provided on request, by the corresponding author.
